# Robotic-assisted surgery as an enabling technology for ovarian-sparing management in pediatric benign ovarian tumours: a comparative study

**DOI:** 10.3389/fped.2026.1880852

**Published:** 2026-06-18

**Authors:** M. M. Cantagalli, Marco Di Mitri, Antonino Morabito, A. Brucculeri, S. Muscolino, E. Severi, E. Bencini, F. Fierro, Enrico Ciardini, Riccardo Coletta

**Affiliations:** 1 School of Pediatric Surgery, University of Florence, Florence, Italy; 2 Department of Pediatric Surgery, Meyer Children’s Hospital, Florence, Italy.; 3 Department of Neurosciences, Psychology, Drug Research and Child Health (Neurofarba), University of Florence, Florence, Italy; 4 Gynecological Unit, Meyer Children’s Hospital IRCCS, Florence, Italy; 5 Radiology Unit, Meyer Children’s Hospital IRCCS, Florence, Italy

**Keywords:** benign ovarian tumour, fertility preservation, minimally invasive surgery, ovarian teratoma, ovarian-sparing surgery, pediatric gynecology, pediatric robotic surgery, robotic-assisted surgery

## Abstract

**Background:**

Preservation of ovarian function is a major objective in the surgical management of benign ovarian tumours in pediatric and adolescent patients. Robotic-assisted surgery may facilitate ovarian-sparing procedures by improving surgical precision and dissection capabilities. This study aimed to evaluate the feasibility and perioperative safety of robotic-assisted ovarian-sparing surgery and to compare outcomes with a pre-robotic cohort.

**Methods:**

A single-centre retrospective comparative cohort study was conducted at a tertiary pediatric referral centre. Consecutive patients aged ≤18 years undergoing surgery for radiologically suspected benign ovarian tumours between January 2023 and December 2025 were included. Patients were divided into a pre-robotic cohort (2023–2024) and a robotic cohort (2025). The primary outcome was the rate of ovarian-sparing surgery. Secondary outcomes included conversion to open surgery, operative time, length of hospital stay (LOS), postoperative complications, reoperation, and postoperative analgesic consumption.

**Results:**

Twenty-four patients were included: 14 in the pre-robotic cohort and 10 in the robotic cohort. Baseline demographic characteristics were comparable between groups. Ovarian-sparing surgery was achieved in all robotic cases (100%) compared with 85.7% in the pre-robotic cohort. No conversions to open surgery occurred in the robotic group, whereas two conversions/oophorectomies occurred in the pre-robotic cohort. Operative time was longer in the robotic cohort (median 2:27:30 vs. 1:42:00; *p*=0.021), while LOS remained comparable. No intraoperative complications or surgical site infections were observed. One robotic patient required reoperation for trocar-site bleeding. Opioid rescue therapy was significantly less frequent in the robotic cohort (10% vs. 50%; *p*=0.048).

**Conclusions:**

Robotic-assisted ovarian-sparing surgery for benign ovarian tumours in pediatric and adolescent patients is feasible and safe. Robotic technology may facilitate fertility-preserving surgery by supporting precise tumour enucleation and consistent preservation of healthy ovarian tissue, particularly in technically demanding cases.

## Introduction

Benign ovarian tumours represent one of the most frequent indications for gynaecologic surgery in paediatric and adolescent patients. Among these, mature teratomas account for the majority of cases, followed by cystadenomas and other benign cystic lesions (1). Although the incidence of malignancy is low in this age group, the clinical presentation often requires surgical management due to symptoms such as abdominal pain, increasing mass size, risk of torsion, or diagnostic uncertainty (2).

When treating ovarian lesions in the paediatric population, the primary surgical objective extends beyond lesion removal. Preservation of healthy ovarian parenchyma is crucial to maintain endocrine function, pubertal development, and future fertility potential (3). Consequently, ovarian-sparing surgery has emerged as the preferred approach whenever oncologically feasible. Nevertheless, achieving complete excision of the lesion while preserving functional ovarian parenchyma remains technically demanding, particularly in the presence of large masses, bilateral disease, or restricted operative workspace (3, 4).

Minimally invasive surgery has progressively replaced laparotomy in the management of benign ovarian masses. Laparoscopy offers well-established benefits, including reduced postoperative pain, shorter hospital stays, faster recovery, and improved cosmetic outcomes. In this scenario, the laparoscopic technique may be demanding due to restricted working space, instrument rigidity, limited degrees of freedom, and the need for precise dissection to avoid unnecessary loss of ovarian tissue. These technical constraints may occasionally lead to conversion to open surgery or to more radical procedures, such as oophorectomy, in situations where the healthy ovarian tissue is not clearly visible (5–7).

Robotic-assisted surgery has emerged as an evolution of minimally invasive techniques, providing three-dimensional high-definition visualisation, tremor filtration, enhanced instrument articulation, and improved ergonomics (8). These technical advantages may facilitate meticulous dissection and reconstruction, potentially supporting a more consistent application of ovarian-sparing strategies (9). While robotic surgery is increasingly utilised in adult gynaecology, its application in paediatric and adolescent populations remains limited, and evidence regarding its clinical impact in benign ovarian pathology is still evolving (10–12).

The aim of this study was to evaluate the feasibility and perioperative safety of robotic-assisted ovarian-sparing surgery for benign ovarian tumours in paediatric and adolescent patients, and to compare outcomes with a pre-robotic cohort to determine whether robotic technology enhances ovarian-sparing management.

## Materials and methods

### Study design and setting

This single-centre retrospective comparative cohort study was conducted at Meyer Children’s Hospital IRCCS, a tertiary paediatric referral centre in Florence, Italy, to evaluate the clinical impact of introducing robotic-assisted surgery for benign ovarian tumours in children and adolescents.

Patients were grouped into two consecutive cohorts based on surgical approach: a pre-robotic period (January 2023–December 2024) and a robotic period (January 2025–December 2025).

The study was approved by the local ethics committee and conducted in accordance with institutional standards for retrospective clinical research. (ROB-PED Study. *Robotic Pediatric Trial (ROB-PED)*. ClinicalTrials.gov Identifier: NCT07438704. Bethesda (MD): National Library of Medicine (US). Available from: https://clinicaltrials.gov/study/NCT07438704?term=NCT07438704&rank=1) (13).

### Patient selection

Patients were identified through a retrospective review of the institutional surgical database and electronic medical records.

Consecutive female patients aged ≤18 years who underwent surgery for a radiologically suspected benign ovarian tumour were identified through a retrospective review of the institutional surgical database and electronic medical records and were included to minimise selection bias.

Inclusion was based on preoperative imaging consistent with benign ovarian pathology. Preoperative assessment included clinical evaluation, ultrasound as the first-line imaging modality, and magnetic resonance imaging in all patients. MRI was used to further characterize the ovarian lesion, evaluate its anatomical relationships, support the preoperative suspicion of benign disease, and guide surgical planning for ovarian-sparing management. Serum tumour markers, including alpha-fetoprotein, beta-human chorionic gonadotropin, lactate dehydrogenase, CA-125, and inhibin B, were requested preoperatively and interpreted together with clinical and radiological findings.

Patients were excluded in case of radiological suspicion of malignancy, emergency procedures, or incomplete perioperative data.

Treatment allocation differed between study periods. During the pre-robotic phase, the surgical approach was determined by surgeon preference and case characteristics. Following the implementation of the robotic platform, robotic-assisted surgery became the standard minimally invasive approach for eligible patients, unless contraindicated.

A minimum follow-up of 30 days was required to evaluate early postoperative outcomes, including complications and reinterventions.

### Surgical approach

During the pre-robotic period, surgical management was performed using laparoscopy or open surgery, depending on tumour size, anatomical characteristics, suspected complexity, and the surgeon’s judgement.

Ovarian-sparing surgery was considered the preferred strategy whenever oncologically appropriate and technically feasible. The procedure consisted of cyst enucleation through careful identification of the cleavage plane between the tumour capsule and normal ovarian parenchyma, aiming to preserve viable ovarian tissue and maintain vascular supply. Oophorectomy was reserved for cases in which complete tumour excision could not be achieved while ensuring adequate residual parenchyma or when intraoperative findings raised concern for compromised ovarian viability.

During the robotic period, all procedures were performed using the da Vinci Xi robotic platform. Port placement was standardised according to patient anthropometry and tumour location to optimise triangulation and instrument reach within the limited abdominal workspace. After docking, cyst enucleation was carried out under three-dimensional high-definition visualisation using articulated robotic instruments. Precise dissection of the tumour capsule was performed along the natural cleavage plane, with meticulous preservation of normal ovarian parenchyma and vascularisation. When necessary, ovarian reconstruction was achieved with fine absorbable sutures to restore anatomical configuration and ensure haemostasis. Attention was paid to minimising thermal spread and mechanical trauma to the residual ovarian tissue.

Across both periods, the surgical objective was complete tumour excision with maximal preservation of functional ovarian tissue, in accordance with fertility-preserving principles.

### Data collection

Demographic, anthropometric, intraoperative, and postoperative data were extracted from electronic medical records. Collected demographic and anthropometric variables included age, weight, height, and body mass index (BMI). Surgical variables comprised histological diagnosis, tumour laterality (right, left, or bilateral), type of procedure (ovarian-sparing surgery or oophorectomy), conversion to open surgery, and operative time. Postoperative outcomes included length of hospital stay (LOS), surgical site infection (SSI), postoperative complications, reoperation within 30 days, and postoperative analgesic consumption. Analgesic use was recorded as the number of administered doses of paracetamol and non-steroidal anti-inflammatory drugs, as well as the requirement for opioid rescue therapy.

Complications were defined as any adverse event occurring within 30 days after surgery. Reoperation was defined as a return to the operating room within 30 days of the index procedure. Pain intensity was assessed using the Visual Analogue Scale (VAS) and recorded as the maximum daily value during the first three postoperative days. Clinical stability was evaluated using the Paediatric Early Warning Score (PEWS), with maximum daily values recorded over the same postoperative interval.

Continuous variables were expressed as median and interquartile range (IQR), whereas categorical variables were reported as absolute numbers and percentages. Given the limited sample size and sparse event counts, effect estimates for key binary outcomes are additionally reported as absolute risk differences and relative risks with 95% confidence intervals (exact binomial confidence intervals for proportions and log-transformed confidence intervals for relative risks). Comparisons between the pre-robotic and robotic cohorts were performed using the Mann–Whitney U test for continuous variables and Fisher's exact test for categorical variables. A two-sided *p*-value < 0.05 was considered statistically significant. All statistical analyses were conducted using R software.

## Results

### Study population

Twenty-four consecutive paediatric and adolescent patients were included, with 14 in the pre-robotic cohort (2023–2024) and 10 in the robotic cohort (2025).

Baseline demographic and anthropometric characteristics were comparable between groups.

Median age was 13.5 years (IQR 11.9–17.1) in the pre-robotic group and 13 years (IQR 12–14) in the robotic group (*p* > 0.05). Median weight was 54.3 kg (IQR 40.5–61.0) vs. 48.9 kg (IQR 43.3–72.0), respectively (*p* > 0.05). Median height was 161 cm (IQR 152–168) in the pre-robotic cohort and 159 cm (IQR 155.5–168.8) in the robotic cohort (*p* > 0.05). Median BMI was 20.1 kg/m^2^ (IQR 16.0–22.9) and 20.0 kg/m^2^ (IQR 19.6–25.0), respectively, with no statistically significant differences (Table 1).

**Table 1:** Baseline demographic and anthropometric characteristics of patients undergoing surgery for benign ovarian tumours in the pre-robotic and robotic cohorts (BMI, body mass index; IQR, interquartile range).

Variable	Pre-robotic (*n* = 14) Median (IQR)	Robotic (*n* = 10) Median (IQR)	*p*-value
Age (years)	13.5 (11.9–17.1)	13 (12–14)	>0.05
Weight (kg)	54.3 (40.5–61.0)	48.9 (43.3–72.0)	>0.05
Height (cm)	161 (152–168)	159 (155.5–168.8)	>0.05
BMI (kg/m^2^)	20.1 (16.0–22.9)	20.0 (19.6–25.0)	>0.05

### Surgical management

**Figure 1:**
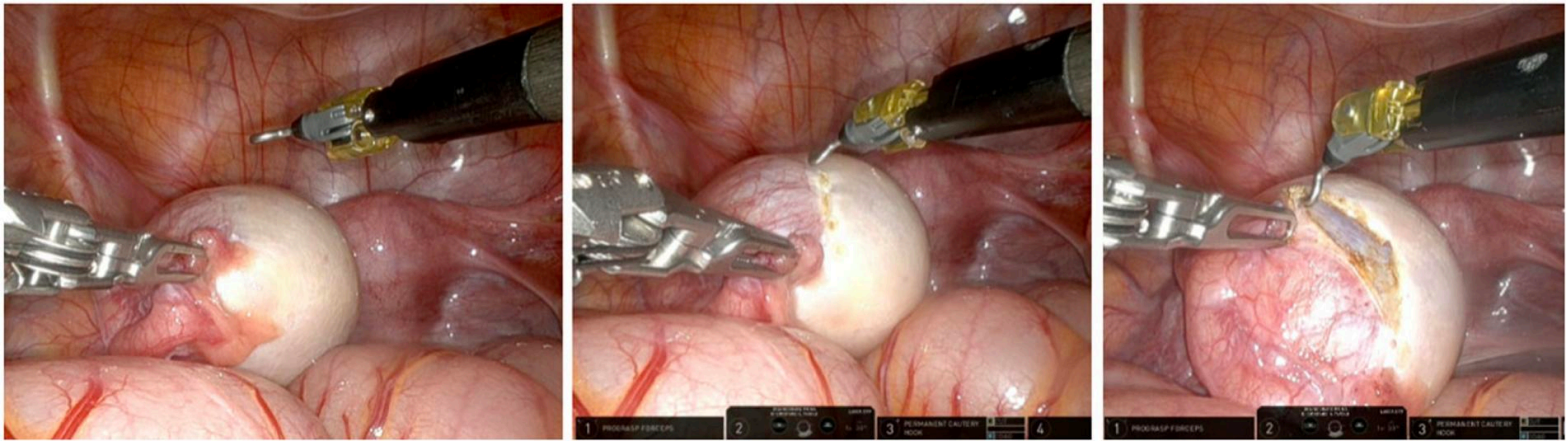
Intraoperative robotic ovarian-sparing surgery for a benign ovarian tumour. The figure illustrates incision of the ovarian albuginea using a monopolar hook and exposure of the cleavage plane prior to tumour enucleation, highlighting the fertility-preserving surgical technique.

Ovarian-sparing surgery was achieved in all patients in the robotic cohort (10/10, 100%, 95% CI 69.2%–100%) compared with 12/14 patients (85.7%, 95% CI 57.2–98.2%) in the pre-robotic cohort, corresponding to an absolute difference of 14.3% (95% CI −10.4% to 39.0%). Two patients (14.3%, 95% CI 1.8–42.8%) in the pre-robotic group required oophorectomy. All procedures in the robotic group were completed with the initially planned minimally invasive approach, with no conversion to laparoscopy or open surgery, yielding a completion rate of 100% (95% CI 69.2%–100%), whereas two patients (14.3%, 95% CI 1.8–42.8%) in the pre-robotic cohort required open surgery or conversion from laparoscopy. No intraoperative complications were recorded in either group (Figure 1; Table 2).

**Table 2:** Comparison of surgical management and ovarian preservation outcomes between the pre-robotic and robotic cohorts (LOS, length of hospital stay; NSAIDs, non-steroidal anti-inflammatory drugs; IQR, interquartile range; SSI, surgical site infection).

Procedure	Pre-robotic (*n* = 14) *n* (%)	Robotic (*n* = 10) *n* (%)
Ovarian-sparing surgery	12 (85.7%)	10 (100%)
Oophorectomy	2 (14.3%)	0 (0%)
Conversion to open	2 (14.3%)	0 (0%)

Histological diagnosis was similar across the cohort, with ovarian teratoma as the predominant diagnosis. In the robotic cohort, 9/10 patients (90%, 95% CI 55.5–99.7%) had teratoma and 1/10 (10%, 95% CI 0.3–44.5%) had cystadenoma.

In the robotic cohort, tumours were right-sided in 6 cases (60%, 95% CI 26.2–87.8%), left-sided in 3 cases (30%, 95% CI 6.7–65.2%), and bilateral in 1 case (10%, 95% CI 0.3–44.5%).

### Operative outcomes

Length of hospital stay was comparable between groups, with a median of 2 days in both cohorts (pre-robotic: IQR 1–2; robotic: IQR 2–3; *p* = 0.084).

Operative time was longer in the robotic group (median 2:27:30, IQR 2:02:00–3:00:00) compared with the pre-robotic cohort (median 1:42:00, IQR 1:23:00–2:03:00), corresponding to a median difference of 45 min (*p* = 0.021). No surgical site infections were observed. A single patient (10%, 95% CI 0.3–44.5%) in the robotic cohort experienced trocar-site bleeding requiring reoperation on postoperative day 1. No further complications or reinterventions occurred. Overall complication rate was low and comparable between groups (0% vs. 10%, *p* = 0.39).

### Postoperative analgesic consumption

Opioid rescue therapy was required significantly less frequently in the robotic cohort (1/10, 10%) compared with the pre-robotic group (7/14, 50%; *p* = 0.048), corresponding to an absolute risk reduction of 40% (95% CI 5.2–74.8%) and a relative risk of 0.20 (95% CI 0.03–1.38).

This reduction occurred despite a higher use of non-opioid analgesics in the robotic group, including paracetamol (median 5 vs. 3 doses; *p* = 0.045) and NSAIDs (median 3 vs. 2 doses; *p* = 0.034) (Table 3).

**Table 3:** Comparison of perioperative outcomes, postoperative complications, and analgesic consumption between the pre-robotic and robotic cohorts (LOS, length of hospital stay; NSAIDs, non-steroidal anti-inflammatory drugs; IQR, interquartile range; SSI, surgical site infection).

Variable	Pre-robotic Median (IQR)	Robotic Median (IQR)	*p*-value
LOS (days)	2 (1–2)	2 (2–3)	0.084
Operative time (hh:mm:ss)	1:42:00 (1:23:00–2:03:00)	2:27:30 (2:02:00–3:00:00)	0.021
Surgical site infection	0 (0%)	0 (0%)	–
Postoperative complications	0 (0%)	1 (10%)	>0.05
Reoperation ≤30 days	0 (0%)	1 (10%)	>0.05
Paracetamol (doses)	3 (2–4)	5 (2–7)	0.045
NSAIDs (doses)	2 (1–3)	3 (3–6)	0.034

### Pain scores and clinical stability

Postoperative pain scores were similar between groups over the first three postoperative days, with no statistically significant differences in median Visual Analog Scale (VAS) values at any time point (day 1: 0.5 vs. 1.0, *p* = 0.75; day 2: 5.0 vs. 4.5, *p* = 0.88; day 3: 3.0 vs. 0.0, *p* = 0.097).

Clinical stability remained comparable between cohorts, with consistently low Paediatric Early Warning Score (PEWS) values throughout the postoperative period.

## Discussion

This study shows that robotic-assisted ovarian-sparing surgery for benign ovarian tumours in children and adolescents is feasible and safe in early experience. Ovarian preservation was achieved in all patients in the robotic cohort, with no conversions to laparotomy and no intraoperative complications.

These findings support the hypothesis that robotic assistance may function as an enabling technology to deliver fertility-preserving surgery more consistently in paediatric patients.

Consistent with previous paediatric robotic gynaecologic series reporting favourable perioperative outcomes (11, 14), our results suggest that robotic assistance may function as an enabling technology to deliver fertility-preserving surgery more consistently (15).

A central finding of this study is the consistency of ovarian preservation achieved with robotic assistance. Fertility and endocrine preservation are key priorities in this population, and benign lesions such as mature teratomas are well recognised as suitable for ovarian-sparing surgery when careful enucleation is performed (16).

In our series, ovarian-sparing surgery was achieved in all patients in the robotic cohort, whereas oophorectomy and conversion to open surgery still occurred in the pre-robotic group. Although the observed increase in ovarian preservation did not reach statistical significance in this limited cohort, it is clinically meaningful, as each avoided oophorectomy contributes to preserving future reproductive potential and reducing the long-term risk of diminished ovarian reserve (17).

Operative time was longer in the robotic group, with a median increase of about 45 min. This is consistent with the early adoption phase of robotic programs, where docking, port placement optimization, and team learning curves may initially extend procedure duration. Importantly, longer operative time did not translate into a longer length of stay or a higher overall complication burden in our series.

Similar trends have been reported in larger comparative studies, where robotic approaches are associated with longer operative times but comparable perioperative safety (18). As experience accumulates, operative efficiency is expected to improve, suggesting that the time difference observed in our series may decrease with programme maturation.

Postoperative outcomes were favourable in both cohorts, with no surgical site infections and a single complication in the robotic group consisting of trocar-site bleeding requiring early reoperation. This highlights that safety in robotic surgery depends not only on intracorporeal precision but also on careful access technique and structured protocols for port placement and closure, particularly in the paediatric setting.

An important finding of this study is the reduction in opioid requirement in the robotic cohort, despite a higher use of non-opioid analgesics. Analgesic consumption showed an interesting pattern: patients in the robotic group received more paracetamol and non-steroidal anti-inflammatory (NSAIDs) drug doses yet were less likely to require opioid rescue therapy. Two explanations may account for this pattern. First, it may reflect an evolution toward multimodal, opioid-sparing analgesic strategies over time. Second, the robotic approach, by enabling more precise dissection and reducing tissue traction and thermal spread, may decrease episodes of breakthrough pain requiring opioid rescue. Pain scores were comparable between groups, indicating that similar perceived pain control was achieved with different analgesic strategies. Given the retrospective design, prospective studies with standardize analgesic protocols and patient-reported outcomes are needed to better define the relationship between surgical platform and postoperative pain control.

From a clinical perspective, these findings contribute to the ongoing debate on the role of robotic platform in paediatric benign adnexal surgery. While laparoscopy provides excellent outcomes and ovarian sparing surgery is increasingly promoted as standard practice (16, 17), the incremental value of robotic assisted surgery may emerge in technically demanding scenarios.

In particular, larger lesions, bilateral disease, or cases requiring precise reconstruction may benefit from enhanced visualisation and instrument articulation. In our series, ovarian preservation was achieved even in a bilateral case in the robotic cohort. Although the sample size does not allow definitive conclusions, these findings support the concept that robotic assistance may function as an enabling technology to deliver fertility-preserving surgery more consistently across varying levels of anatomical complexity.

## Limitations

This study has several limitations. First, its retrospective design and relatively small sample size limit statistical power, particularly for uncommon outcomes, and may affect generalisability.

Second, comparing two consecutive time periods introduces the potential for temporal confounding, including changes in surgical experience, perioperative management, and postoperative analgesic protocols.

Third, follow-up was limited to early postoperative outcomes, precluding assessment of long-term endpoints, including ovarian function, recurrence, menstrual regularity, ovarian reserve, and fertility. Long-term fertility data are not currently available due to the retrospective design of the study, the young age of the patients, and the relatively short follow-up period. Future studies should include longitudinal assessment of ovarian function, ovarian reserve markers, menstrual outcomes, recurrence, and reproductive outcomes.

Finally, Prospective multicentre studies with larger cohorts and standardised perioperative protocols are warranted to better define the role of robotic-assisted surgery in fertility-preserving management of benign ovarian tumours in paediatric patients.

## Conclusions

Robotic-assisted ovarian-sparing surgery for benign ovarian tumours in paediatric and adolescent patients is feasible and safe in early experience.

The robotic platform appears to serve as an enabling technology, facilitating precise tumour enucleation and supporting a more consistent ovarian-sparing strategy, particularly in technically demanding cases.

These findings suggest that robotic assistance may increase the likelihood of ovarian preservation while reducing the need for more radical procedures. Larger prospective studies are warranted to confirm these observations and assess long-term functional outcomes.

## Data Availability

The raw data supporting the conclusions of this article will be made available by the authors, without undue reservation.
